# Myocardial strain analysis using cardiac magnetic resonance in patients with calpainopathy

**DOI:** 10.1186/s13023-021-01826-0

**Published:** 2021-04-30

**Authors:** Silvio Quick, Max Winkler, Uwe Speiser, Karim Ibrahim, Jochen Schäfer, Axel Linke, Kun Zhang, Marian Christoph, Felix M. Heidrich

**Affiliations:** 1 Department of Cardiology, Angiology and Intensive Care, Klinikum Chemnitz gGmbH, Medizincampus Chemnitz der Technischen Universität Dresden, Chemnitz, Germany; 2 Department of Internal Medicine and Cardiology, Herzzentrum Dresden Universitätsklinik, Technische Universität Dresden, Dresden, Germany; 3 Department of Neurology, Universitätsklinikum Carl Gustav Carus, Technische Universität Dresden, Dresden, Germany; 4 Department of Internal Medicine and Cardiology, Charité – Universitätsmedizin Berlin, Campus Virchow-Klinikum, Berlin, Germany

**Keywords:** Calpainopathy, Limb-girdle muscular dystrophy, LGMD2A, Cardiac magnetic resonance imaging, Strain analysis, Feature-tracking, Global longitudinal strain

## Abstract

**Background:**

Limb–girdle muscular dystrophy (LGMD) is a genetically and clinically heterogeneous group of rare muscular dystrophies. Subtype 2A (LGMD2A) also known as “calpainopathy” is an inherited autosomal recessive gene defect. Cardiac dysfunction is common in several forms of LGMD. Cardiac involvement in LGMD2A, however, is not clear. The aim of this study was to perform cardiac magnetic resonance (CMR)-based strain analysis in LGMD2A patients, as this is a diagnostic parameter of subclinical cardiac involvement and a powerful independent predictor of mortality. We conducted the largest prospective cardiac magnetic resonance study to date, including 11 genetically verified LGMD2A patients and 11 age- and sex-matched control subjects and performed CMR-based strain analysis of the left and right ventricles.

**Results:**

Left and right global longitudinal strain (GLS) were not significantly different between the two groups and within normal reference ranges (left ventricle: control − 21.8 (5.1) % vs. patients − 22.3 (3.2) %, *p* = 0.38; right ventricle: control − 26.3 (7.2) % vs. patients − 26.8 (5.8) %, *p* = 0.85). Also, global circumferential and radial strains did not significantly differ between the two groups (*p* = 0.95 and *p* = 0.86, respectively). LGMD2A patients did not show relevant amounts of late gadolinium enhancement (LGE) or malignant ventricular arrhythmias.

**Conclusions:**

No evidence of even subtle cardiac dysfunction is evident form CMR-based strain analysis in LGMD2A patients. Malignant ventricular arrhythmias were not detected. Thus, in case of non-pathological initial echocardiographic and electrocardiographic examination, a less frequent or even no cardiac follow-up may be acceptable in these patients. However, if there are signs and symptoms that suggest an underlying cardiac condition (e.g. palpitations, angina, shortness of breath), this approach needs to be individualized to account for the unknown.

## Background

Limb-girdle muscular dystrophy (LGMD) is a group of inherited autosomal disorders characterized by progressive muscle weakness in the scapular and pelvic girdle and trunk muscles. Subtype 2A (LGMD2A, “calpainopathy”) is the most common form of LGMD in European countries, affecting about 1:100,000 people [1, 2]. It also is the most common neuromuscular autosomal recessive disorder after spinal muscular atrophy. LGMD2A is caused by mutations in the calpain 3 gene (CAPN3), encoding for a muscle‐specific proteolytic enzyme, calpain‐3 [3]. Calpain-deficiency has been shown to cause sarcomere abnormalities and it leads to muscle fiber death, whereas its functional impairment affects muscle membrane remodeling and repair [4].

In several forms of LGMD, cardiac dysfunction occurs, which may manifest as hypertrophic or dilated cardiomyopathy and cardiac arrhythmias. Significant cardiac involvement has been documented frequently in LGMD2C-F, 2I, and LGMD1B forms of the disease, rarely in the LGMD1C and 2B subtypes [5, 6].

Attention to cardiac involvement in patients with muscular dystrophies is increasing, probably due to an increased molecular genetic knowledge of the different types of muscular dystrophies. As patients live longer, heart failure and arrhythmias contribute more to mortality, stressing the need for more knowledge of the degree and progression of cardiac involvement in these patients.

To date, evidence of cardiac involvement in calpainopathy (LGMD2A) is not clear. An echocardiography study of 14 LGMD2A patients showed no relevant cardiac dysfunction [7]. However, multiple case reports and one larger study of 52 patients have suggested cardiac involvement including heart rhythm disturbances and left ventricular dysfunction [8–11].

Standard advocated cardiac screening methods (electrocardiography and echocardiography) are often unremarkable and cannot detect and quantify myocardial damage at early stages of disease.

Strain analysis in cardiac magnetic resonance imaging (CMR) is a new promising tool to capture subtle alterations that result from early disease stages of the myocardium when other parameters such as ejection fraction are still unremarkable. Even though it is a relatively rapid procedure, it is not yet part of routine CMR protocols in clinical practice. In recent studies, CMR based strain analysis has been shown to be a valuable marker for risk prediction. Global longitudinal strain (GLS) was associated with increased all-cause mortality as well as increased death in patients with dilated cardiomyopathy [12, 13]. Moreover, Romano et al. showed in a large multicenter study that GLS derived from CMR is a powerful independent predictor of mortality in patients with even preserved ejection fraction [14].

## Methods

### Aim and design of the study

The aim of the present study was to perform strain analysis in LGMD2A patients using CMR for the first time. We examined left and right ventricular function with strain analysis to detect subtle early myocardial functional changes. As this study was not designed to provide outcome data, we want to discuss our results in light of the current literature and gather information on the risk of heart failure and sudden cardiac death in LGMD2A patients.

Thirteen patients with genetically verified LGMD2A were screened for this study. CMR image quality for myocardial strain analysis was sufficient in 11 patients. The diagnosis of calpainopathy was established according to the standard reported by Fanin et al. [15] DNA analyses were performed in all patients. All 11 patients had mutations in the CAPN3 gene. Three patients had one mutation in one allele, and 8 patients of 7 families had two mutations (1 mutation in each allele, 5 compound heterozygous patients, 3 patients were homozygous for mutations in CAPN3; Table 1).

**Table 1:** Baseline clinical characteristics, genotype, severity of symptoms in patients with limb-girdle muscular dystrophy type 2A

Patient	Age at presentation	Gender	Mutation	Walking ability	Scapular winging	Joint contraction	Co-morbidities	Family history	Serum CK
1	25	♀	HE*, Exon 21: c.2242C>T	Ambulant	Bilateral	Achilles	No	Yes	32
2	48	♂	HE*, Exon 21: c.2242C>T	Ambulant	Bilateral	Achilles	No	No	7
3	55	♀	CH, Exon 04: c.550delA Exon 20: c.2162G>A	Ambulant	No	No	No	No	14
4	32	♀	HO, Exon 04: c.550delA	Ambulant	Bilateral	Achilles	No	No	9
5	28	♂	CH, Exon 04: c.550delA Exon17: c.1992+1G>T	Wheelchair- bounded	Bilateral	Achilles	No	Yes	17
6	25	♀	CH, Exon 04: c.550delA Exon 24: c.2440-3C>G	Wheelchair- bounded	Bilateral	Achilles	No	No	22
7	51	♀	HO,Exon11:c.1524+3C>G	Wheelchair-bounded	Bilateral	Achilles	AFIB	No	5
8	31	♂	CH,Exon:04:c.del598_612 Exon 10: c.1303G>A	Ambulant	No	No	No	Yes	54
9	23	♀	CH, Exon 04: c.550delA Exon 17: c.1992+1G>T	Ambulant	Bilateral	Achilles	No	Yes	24
10	27	♂	HO,Exon 04: c.550delA	Ambulant	No	No	No	No	8
11	55	♀	HE*, Exon 04: c.550delA	Ambulant	No	No	No	No	10

^*^No second mutation detectable

HE, heterozygous; HO, homozygous, CH, compound heterozygous; CK, creatine kinase (µmol/(s*L); AFIB, atrial fibrillation

We selected 11 age- and sex-matched controls from our database. Cardiac disease was ruled out in control subjects based on results from clinical examination, echocardiography, CMR and laboratory data. Written informed consent was obtained from all participants. The study protocol conforms to the ethical guidelines of the 1975 Declaration of Helsinki.

### Cardiac magnetic resonance imaging and strain analysis

We worked with a 3.0 T magnetic resonance imaging system (Signa HDxt 3.0 T, General Electric, Milwaukee, USA) using an 8-channel cardiac coil and prospective electrocardiographic R-wave triggering. Real-time scout images in axial, sagittal, and coronal planes were used to localize the cardiac position within the thorax. From ventricular apex to base, ECG-triggered, breath-hold, balanced steady-state free precession sequences (SSFP) were obtained in the short axis, 2-chamber, and 4-chamber view to display cardiac function. Analysis of left ventricular global radial, longitudinal, and circumferential, as well as right ventricular global longitudinal 2D strain values were obtained using a Feature Tracking Software (Medis Suite MR, Medis Medical Imaging Systems). All patients underwent a late gadolinium enhancement imaging protocol using a segmented inversion-recovery pulse sequence starting 10 to 15  min after a weight-based injection (cumulative dose 0.15  mmol/kg) of gadolinium diethylenetriamine pentaacetic acid (Magnevist®, Bayer HealthCare Pharmaceuticals Inc., Berlin, Germany). Regional fibrosis was identified by LGE within the myocardium, defined quantitatively by myocardial postcontrast signal intensity 2 SDs above that within a reference region of remote myocardium within the same slice. Extent of LGE was calculated after manual tracing of endocardial and epicardial borders on each short-axis slice. LGE volume was expressed as a percentage of total myocardial mass.

### 24 h electrocardiography

An ambulatory electrocardiograph (ECG, CardioMem® CM 3000, Getemed) was used to detect ventricular ectopic beats (VEBs), and disturbance of rhythm (arrhythmia, tachycardia, bradycardia). The patients had to wear the ambulatory electrocardiograph for 24 h while continuing their daily activities.

### Statistical analysis

SPSS 20.0 (SPSS, Inc., Chicago, IL, USA) was used for statistical analyses. Data are presented as mean (SD), median (IQR), or n (%) unless otherwise stated. For group comparisons, independent samples t-test and Wilcoxon signed-rank test were used. Pearson’s coefficient was used for correlation analysis. A *p* value <0.05 was considered statistically significant.

## Results

### Baseline characteristics

An overview of the baseline demographic, clinical data and co-morbidities of the patient population are shown in Table 1. Mean age was 39.3 (10.8) years for controls and 36.4 (13) years for LGMD2A patients, evenly distributed between male and female. Of the 11 LGMD2A patients (4 men, 7 women), six patients had initial weakness of the pelvic girdle muscles, which gradually progressed to a typical limb-girdle pattern. Scapular winging and achilles tendon contracture were found in 7 patients (63%). Mean serum creatine kinase level was 18.4 µmol/(s*L). None of the patients had symptoms of heart failure, e.g. shortness of breath, oedema, syncope, angina pectoris or palpitations.

### Left and right ventricular CMR parameters

Left and right ventricular CMR parameters of LGMD2A patients and control subjects are shown in Table 2. Left and right ventricular global strain parameters did not significantly differ between the two groups. Of note, none of the patients showed an average GLS of > − 16%. In addition, ejection fraction (EF) of both ventricles, right ventricular fractional area change (RVFAC) and tricuspid annular plane systolic excursion (TAPSE) were not significantly different between controls and patients.

**Table 2:** Myocardial strain and CMR characteristics of patients and controls

	LGMDA2 patients (n = 11)	Controls (n = 11)	*p* value
Left ventricular parameters			
GLS, %	− 22.3 (3.2)	− 21.8 (5.1)	0.38
GRS, %	50.9 (17)	54.3 (12)	0.59
GCS, %	− 29.8 (5.2)	− 30 (4.8)	0.86
LVEF, %	55.9 (2.6)	60.62 (6.6)	0.15
LVEDV, ml	102.5 (17.4)	155.4.0 (29.1)	< **0.001**
LV mass, g	77.7 (17.2)	112.9 (44.3)	**0.04**
LGE, %	0.4 (0.8)	–	
Right ventricular parameters			
GLS %	− 26.8 (5.8)	− 26.3 (7.2)	0.85
GLS free wall, %	− 32.3 (5.6)	− 32.7 (7.9)	0.91
GLS septum %	− 21.3 (5.6)	− 19.9 (6.6)	0.64
RVEF, %	63.5 (5.8)	60 (6.8)	0.34
RVFAC, %	46.3 (9)	48 (8)	0.66
TAPSE, mm	23 (6.1)	28.5 (5)	0.09
RVEDV, ml	88.5 (19.8)	142.7 (38.4)	**0.01**

Bold indicates significant between-group differences

Data are presented as mean (SD)

GLS, global longitudinal strain; GRS, global radial strain; GCS, global circumferential strain; LVEF, left ventricular ejection fraction, LVEDV, left ventriclar end-diastolic volume; RVEF, right ventricular ejection fraction; RVFAC, right ventricular fractional area change; TAPSE, tricuspid annular plane systolic excursion; RVEDV, right ventricular end-diastolic volume; LGMD2A, limb girdle muscular dystrophy

LGMD2A patients had smaller left and right end-diastolic volumes and lower LV mass. In one patient a small amount (2% of LV-mass) of late gadolinium enhancement was found, located at the insertion areas of the right ventricle (RV) at the anterior ventricular septum.

### 24-h electrocardiographic recording

Heart rhythm disturbances were seen in 1 LGMD2A patient, who suffered from atrial fibrillation. Ventricular ectopic beats (VEB) were detected in 3 of 11 patients (27%). On average, 11.5 VEB (range 0;113) were seen in 24 h. However, we could not detect any non-sustained or sustained fast malignant ventricular arrhythmias. Supraventricular ectopic beats (SVEB) were found in 5 of 11 patients (45%). However, average number of SVEB was small (5.5, range 0;58).

## Discussion

This is the first CMR-based strain analysis study of patients suffering from calpainopathy. Moreover, to date it is the largest prospective imaging study evaluating cardiac manifestation of calpainopathy. Our results show that left and right ventricular myocardial strain parameters did not significantly differ between LGMD2A patients and control subjects and were within the normal range. Furthermore, we could not detect malignant ventricular arrhythmias in LGMD2A patients.

LGMD2 comprises 17 genetically heterogenous subtypes (LGMD2A–Q) [16]. The pathophysiologic mechanism behind seems to be caused by dysfunctional proteins at several different levels of the muscle cell. This leads to changes in intra- and extracellular enzyme activities and disturbed signal transduction across the plasma membrane, resulting in degeneration and necrosis of skeletal myofibers and—in some subtypes of the disease—cardiomyocytes with gradual replacement by fat- and fibrotic tissue [17]. Serum-CK elevation is thought to be a reflection of this chronic process. In LGMD2A patients presenting with increased CK levels, eosinophilic myositis has been shown.

Cardiac involvement is frequently observed in LGMD2 subtypes, including cardiomyopathy and cardiac arrhythmias [7]. Multiple case reports have suggested cardiac involvement, for example heart rhythm disturbances and left ventricular dysfunction [8–10]. On the other hand, an echocardiography study of 14 LGMD2A patients could not show relevant cardiac dysfunction [7]. However, echocardiography might only serve as screening tool to detect overt cardiac dysfunction. Due to limited cardiac tissue characterization and limitations in volumetric measurements, especially of the right ventricle, echocardiography is not suitable for the detection of subtle and/or subclinical cardiac dysfunction.

Cardiovascular magnetic resonance (CMR) can overcome this problem. CMR is a precise and reliable method for assessing global and regional cardiac function and is the best noninvasive method for tissue characterization including myocardial fibrosis caused by ischemic or nonischemic disease [18, 19].

To further evaluate subtle cardiac changes in our group of clinically asymptomatic patients, in the present study we used the evolving method of CMR-based strain analysis, also referred to as “feature-tracking” [20]. This method provides detailed information on global and regional active ventricular deformation. Subtle changes in the structure and geometry of the LV and RV myocardium may lead to changes in LV/RV deformation that may not be detectable calculating LV/RV ejection fractions. Especially the determination of GLS is of additional prognostic value because a worsening of GLS has been associated with higher mortality even in patients with preserved ejection fraction [12–14, 21–24]. In a recent study by Fröjdh et al. of consecutive adult patients who were referred to their center for standard CMR, a GLS > − 16% was associated with a higher mortality [25]. None of our patients had a GLS > − 16%. Furthermore, tissue characterization did not detect significant amounts of LGE as a marker of myocardial fibrosis. LGE is an excellent prognostic parameter. It is associated with an increased risk of all-cause mortality, heart failure hospitalization, and sudden cardiac death [26]. LGE was only present in one LGMD2A patient. However, this was located at the insertion area of the right ventricle in the anterior septum. RV insertion LGE has been characterized histologically as benign spatial expansion of the extracellular matrix created by the arrangement of intersecting myocardial fibers (rather than prognostically relevant replacement fibrosis) and this is common even in healthy subjects [27].

Three of the eleven LGMD2A patients showed a low count of VEBs. We do not interpret these as a disease specific cardiac manifestation of LGMD2A. In fact, VEBs are common and have been described in 40–69% of healthy low-risk individuals as detected by 24-h ambulatory ECG recordings [28, 29]. In patients with underlying structural heart disease, VEBs can trigger ventricular arrhythmia, but in individuals with structurally normal hearts they are often considered a benign process that does not require treatment or intervention [33, 34].

Taken together we conclude that even subclinical structural heart disease was not present in our LGMD2A patient cohort. One explanation might be that there is absence of calpain 3 protein expression in adult cardiomyocytes, despite CAPN3 mRNA transcripts being present [30]. Transcripts of CAPN3 appear in the early embryonic heart during human development. Transcripts are initially seen in all heart compartments. Later, they become restricted to the atrium during the embryonic period. During fetal development, CAPN3 transcripts eventually also disappear from there. However, the evidence of transcriptional activity does not imply that the corresponding proteins are expressed [7, 30].

The discrepancy between our data and the proposed cardiac manifestations in LGMD2A patients reported in the case reports cited above are obvious. In a study of 68 LGMDR1/LGMDA2 patients, one out of 52 patients (1.9%) showed signs of dilated cardiomyopathy [11]. In Germany in 2006 the prevalence of heart failure was 1.6% in women and 1.8% in men, with numbers increasing considerably with advancing age [31]. Therefore, these patients may have heart failure not because of but in addition to LGMD2A (for example due to ischemic cardiomyopathy, past myocarditis, or primary dilated cardiomyopathy).

LGMD2A patients had smaller LV and RV end-diastolic volumes and LV mass compared to control subjects. We consider this not a pathological finding, as most of our patients had limited physical mobility. LV volume and mass rapidly change in response to physical conditioning and deconditioning [32].

## Limitations

1. This study only included 11 patients. However, calpainopathy is a quite rare disease and a myocardial strain analysis of a cohort of 11 patients has never been done before.
2. With a mean age of 39 years, our patients represent a relatively young patient cohort. It is possible that subclinical manifestations may become structurally and clinically overt with increasing age.
3. Because myocardial tissue characterization was not the scope of this study, we did not use T1 and T2 CMR mapping sequences for tissue characterization and neither were endomyocardial biopsy samples obtained.
4. Conclusions drawn on rhythm disturbances are based on 24-h ambulatory electrocardiographs, only resembling a snapshot
5. Our patient cohort represents only a few of the more than one hundred different calpain 3 gene mutations causing LGMD2A. However, we think that our results can be extrapolated to other calpain 3 gene mutations due to the concept that there is absence of calpain 3 protein expression in adult cardiomyocytes.

## Conclusion

LGMD2A patients did not show signs of even subtle cardiac involvement using CMR-based strain analysis. Thus, in the case of non-pathological initial echocardiographic and electrocardiographic examinations, a less frequent or even no cardiac follow-up maybe acceptable in these patients, as was previously suggested by Sveen et al. [7] However, if there are signs and symptoms that would suggest an underlying cardiac condition (e.g. palpitations, angina, shortness of breath), this approach needs to be individualized to account for the unknown.

## Data Availability

The datasets used and/or analysed during the current study are available from the corresponding author on reasonable request.
